# Biomechanical assessment of the maxillary position after Le Fort I osteotomy using rapidly biodegradable miniplates and screws: an experimental study

**DOI:** 10.7150/ijms.118208

**Published:** 2026-01-01

**Authors:** Shintaro Sukegawa, Rei Hanai, Fumi Nakai, Yasuhiro Nakai, Masato Saika, Minoru Miyake

**Affiliations:** Department of Oral and Maxillofacial Surgery, Kagawa University Faculty of Medicine, 1750-1, Ikenobe, Miki-cho, Kita-gun, Kagawa 761-0793, Japan.

**Keywords:** orthognathic surgery, biomechanical loading evaluation, resorbable plate, Le Fort I osteotomy

## Abstract

Absorbable plates have become increasingly common in orthognathic surgery. Multiple plate types are available, including poly-L-lactic acid (PLLA) and PLLA/polyglycolic acid (PGA), the latter of which is expected to be absorbed more rapidly. However, the application of these materials in Le Fort I osteotomy is not clearly established. In this study, the strength and biomechanical properties of PLLA and PGA-co-PLLA absorbable plates were compared using an in vitro LeFort I osteotomy model. Basic physical strength was evaluated using tensile, bending, indentation, and handling test s. Biomechanical evaluations using the Le Fort I osteotomy model included anterior (with 0, 3, and 5 mm anterior movement) and occlusal (with a 2 mm downward vertical movement) indentation tests. In terms of basic physical properties, PLLA showed significantly higher strength than that of PGA-co-PLLA in tensile, bending, and indentation tests. In handling tests, PGA-co-PLLA demonstrated superior performance. In biomechanical evaluations, no significant differences were observed between PLLA and PGA-co-PLLA in anterior or occlusal indentation testing. Despite the greater basic physical strength of PLLA than PGA-co-PLLA, there were no significant differences between the two plate types in the biomechanical evaluation of Le Fort I osteotomy. These findings provide a basis for plate selection in clinical practice.

## Introduction

Le Fort I (LF1) osteotomy is a common orthognathic surgery 1,2. Masticatory function is achieved by synchronized movements of anatomical structures in the maxillofacial region. The maxilla is an immovable bone fixed at the base of the skull, which receives force from the moving mandible. Therefore, stable maxillary fixation is required for orthognathic surgery.

Absorbable plates have become increasingly common in the field of oral surgery in recent years. A major advantage of absorbability is that re-operation is unnecessary, which reduces medical costs. Various characteristics can be achieved by adding materials to basic poly-L-lactic acid (PLLA) 3. For example, the times required for absorption and decomposition can be shortened by incorporating polyglycolic acid (PGA). This is a great advantage when used in palpable areas of the maxillofacial region, or in cases where there is a risk of complications 4. However, the strength depends on the ratio of materials used.

The breakdown of internal fixation and effects of the plate on occlusion are concerns for clinicians. To address these problems, several researchers have investigated bioabsorbable fixation techniques using unique biomechanical methods 5-7. However, most studies have focused on the mandible, and detailed analyses of bone union following LF I osteotomy are lacking. Additionally, the lack of comparative analyses of biomechanical strength of various absorbable materials has led to some controversy surrounding plate selection. The early degradation of PGA-co-PLLA offers several clinical advantages. Rapid absorption minimizes the long-term presence of foreign material and reduces the risk of late-onset inflammatory or foreign body reactions. Moreover, it helps to prevent the sensation of a residual foreign body perceived by the patient 8, which is particularly beneficial in the maxillofacial region. Therefore, PGA-co-PLLA can be considered a useful option in cases where long-term mechanical support is not required.

This study aimed to compare the strength and biomechanical properties of PLLA mono-material plates and PGA-co-PLLA absorbable plates when applied to LFI osteotomies using an in vitro model.

## Materials and Methods

### Materials

Two different absorbable osteosynthesis systems were used: GrandFix®, made of PLLA monomaterial, and NEOFIX-R®, made of PGA-co-PLLA at a ratio of 82:18 (both manufactured by GUNZE MEDICAL LIMITED., Ltd., Osaka, Japan). All plates and screws were fabricated within the same period of time (i.e., within 2 weeks).

### Evaluation of basic physical properties of plates

#### Sample preparation and measurement

**Figure 1:**
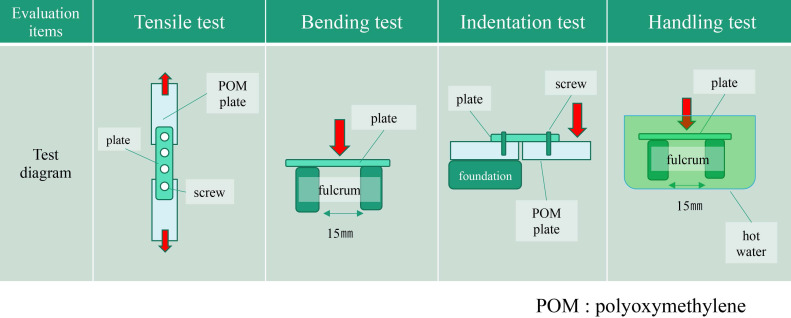
Summary of basic physical properties of plates.

Two types of absorbable plates were fixed to a 3-mm-thick polyoxymethylene (POM) plate with their respective absorbable screws. The basic physical properties of the plates were evaluated using bending, tensile, indentation, and handling tests (Figure 1).

Each absorbable bone material consisted of a 1.5-mm-thick plate and a screw (diameter 2.2 mm, length 5 mm). The experimental fixation model was attached to an Autograph AG-20kNXD test frame (Shimadzu Corporation, Kyoto, Japan) using a chuck. The maximum stress and stress at 1 mm displacement were measured, and the load was applied at 10 mm/min. Each of the three strength tests (tensile, bending, and indentation) was conducted 8 times, and the mean value was calculated from the six values obtained after removing the maximum and minimum values.

To evaluate operability, a plate was placed on a fulcrum in water, the center of the plate was pushed at a speed of 2.0 mm/min with a fulcrum distance of 15 mm, and the bending strength was measured. The water bath was set at various temperatures (37 °C, 60 °C, 70 °C, and 80 °C), and the bending test jig and plate were immersed for 10 s. Bending strength was measured at each temperature.

### Biomechanical loading test

#### Sample preparation

A maxillary model-pressing test was performed on the anterior and occlusal planes as a biomechanical load test.

LF1 osteotomy was performed by creating a jig and performing a standard osteotomy from the piriform rim to the pterygomaxillary suture. All fixations were performed using standard four-hole, 1.5-mm-thick L-shaped absorbable plates fixed with 2.2-mm screws to the piriform rim and zygomatic buttress on both sides for a total of four plates.

Models with different degrees of maxillary movement were created. For the biomechanical load test in the anterior direction, models with maxillary bone movements of 0, 3, and 5 mm were prepared.

**Figure 2:**
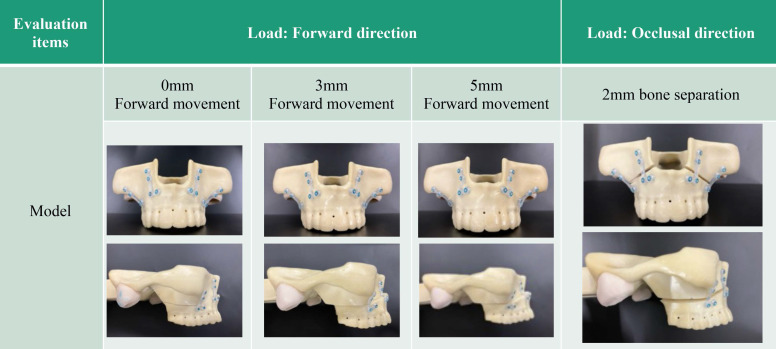
Models for biomechanical loading.

In addition, a model with a 2 mm gap between the bones was prepared for the biomechanical load test of the occlusal surface (Figure 2).

#### Biomechanical loading test method

The model was attached to a testing machine based on a biomechanical load model (AG-20KNX; Shimadzu Corporation). The fit of the model to the machine was ensured using a metal support.

**Figure 3:**
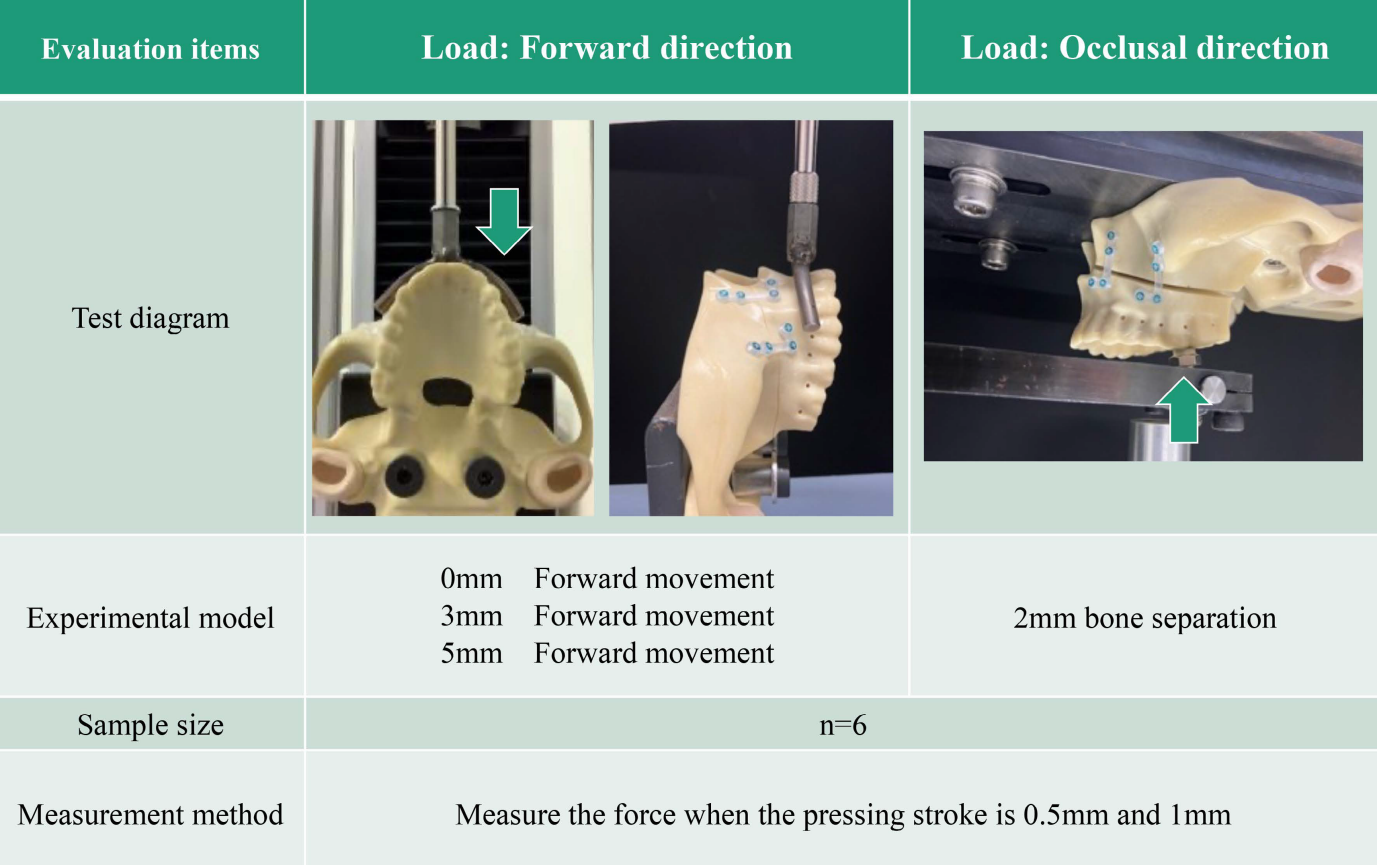
Summary of biomechanical loading test.

A biomechanical load test in the anterior direction was performed according to a previously described biomechanical evaluation method 9. This test was designed to evaluate fixation stability in the anterior maxillary region after Le Fort I osteotomy and to simulate postoperative stresses acting on the anterior maxilla caused by the tension and compression of the upper lip and perioral soft tissues, rather than occlusal loading during mastication. A horseshoe-shaped arm was created to apply a force to the anterior maxilla. Linear displacement was created at a speed of 1 mm/min, and the indentation strengths at 0.5 mm and 1 mm were compared (Figure 3). The left first molar was used as the loading point for the indentation test of the maxillary biomechanical model on the occlusal surface.

In both tests, a preload was applied to each specimen at the beginning and the load was readjusted to zero at the start of the test. The slope was obtained for every 0.005 mm of stroke up to 4 mm and a force of 50 N. The position where the slope changed at one point was used as the inflection point, and correction was performed.

### Statistical analysis

During the tests, load and displacement data were recorded digitally and summarized in an electronic database using Microsoft Excel (Microsoft, Redmond, WA, USA). The digital database was transferred to JMP PRO ver. 16.1.0 for Macintosh (SAS Institute, Cary, NC, USA) for statistical analyses. The groups for each plate were compared using the Mann-Whitney U test. Statistical significance was defined as p < 0.05.

## Results

### Basic physical properties of plates

**Figure 4:**
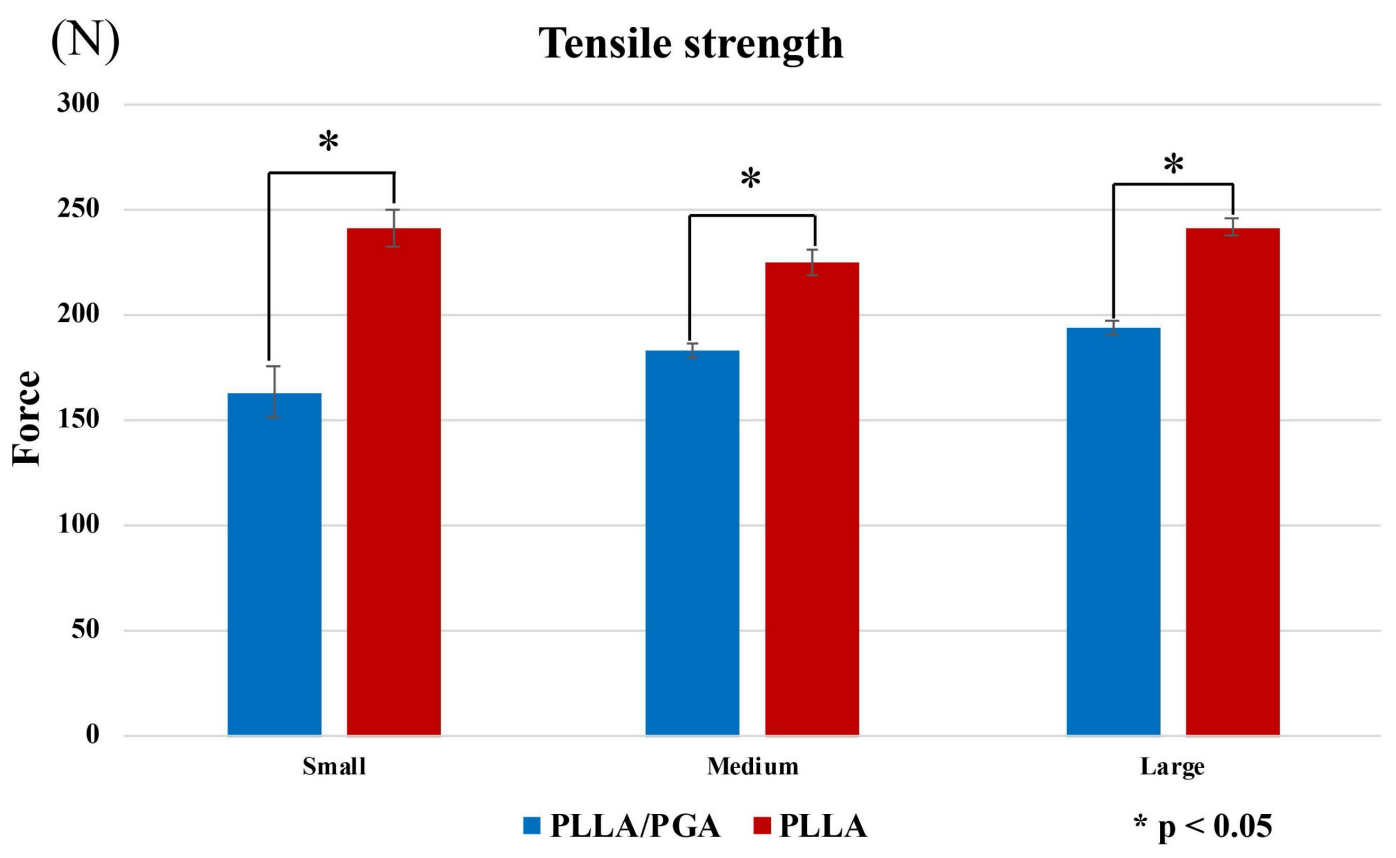
Comparison of PGA-co-PLLA and PLLA in tensile strength tests.

In the tensile test, PGA-co-PLLA was significantly weaker than PLLA for small, medium, and large plates (P < 0.001). There were no differences in characteristics with respect to size, and the small plate showed the greatest differences between PGA-co-PLLA and PLLA (i.e., strengths were 68.0%, 81.4%, and 80.4% of that of PLLA for small, medium, and large plates, respectively) (Figure 4).

**Figure 5:**
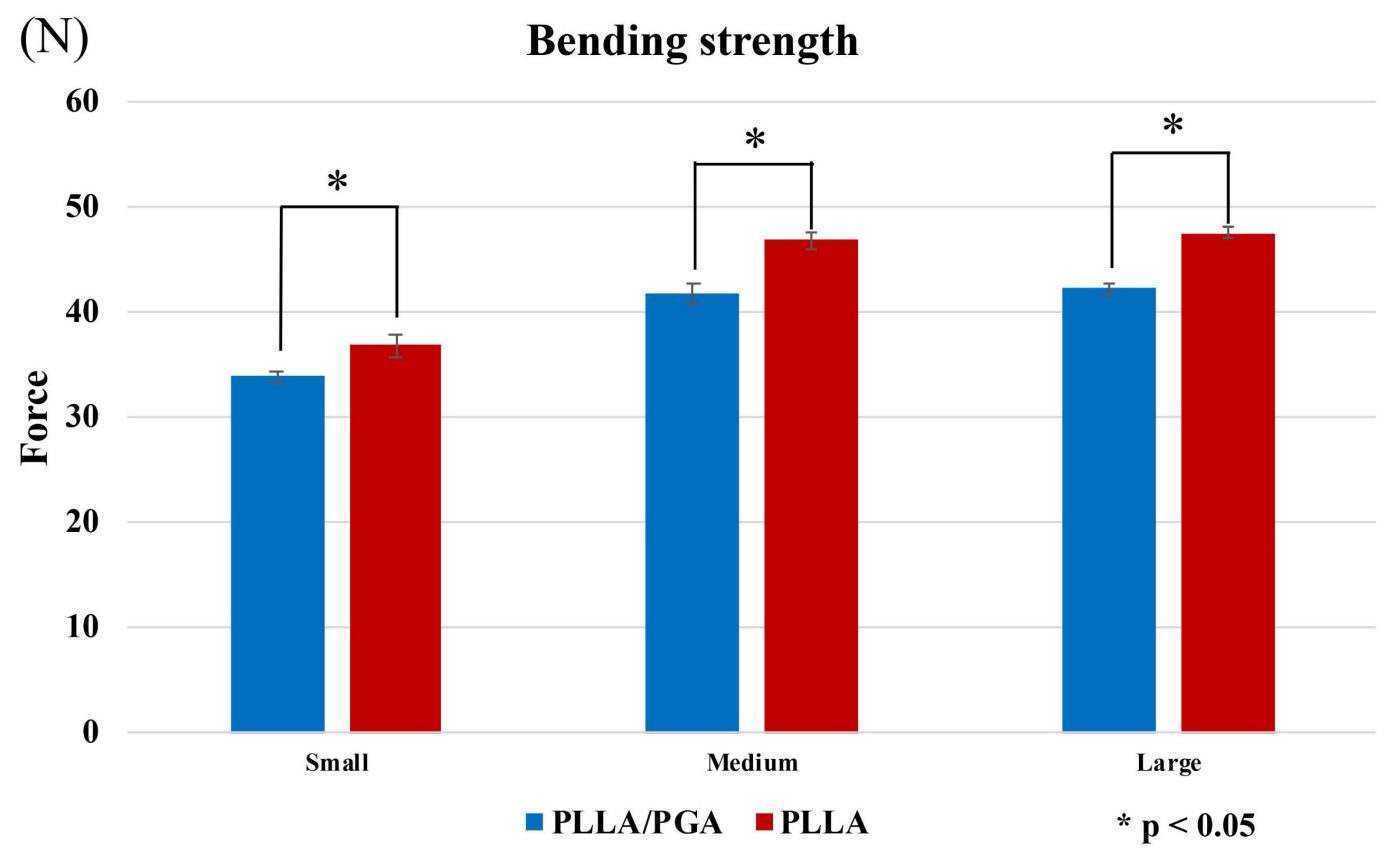
Comparison of PGA-co-PLLA and PLLA in bending strength tests.

In the bending tests, PGA-co-PLLA was significantly weaker than PLLA for the small, medium, and large plates (P < 0.001). The difference in strength was lower for small plates than for medium and large plates ((PGA-co-PLLA)/(PLLA) small: 92.1%; medium: 89.2%; large: 89.0%) (Figure 5).

**Figure 6:**
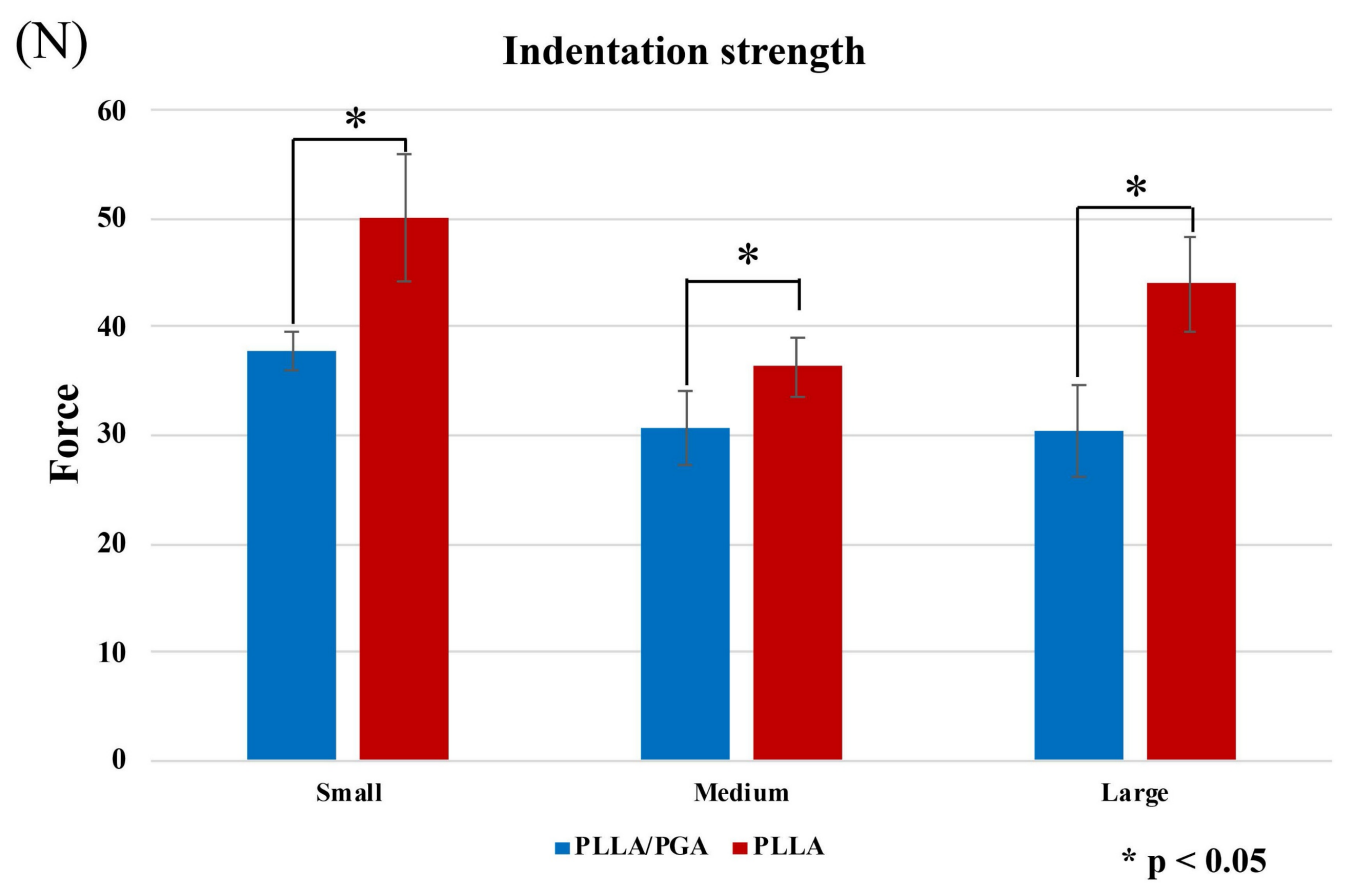
Comparison of PGA-co-PLLA and PLLA in indentation strength tests.

In the indentation strength test, PGA-co-PLLA was significantly weaker than PLLA for small, medium, and large plates (P < 0.001). Results did not differ with respect to plate size; the largest difference was observed for the large plate, where PGA-co-PLLA had 69.2% of the strength of PLLA (small, 75.4%; medium, 84.5%) (Figure 6).

These findings indicate that PLLA was significantly stronger than PGA-co-PLLA in terms of bending, tensile, and indentation strengths.

**Figure 7:**
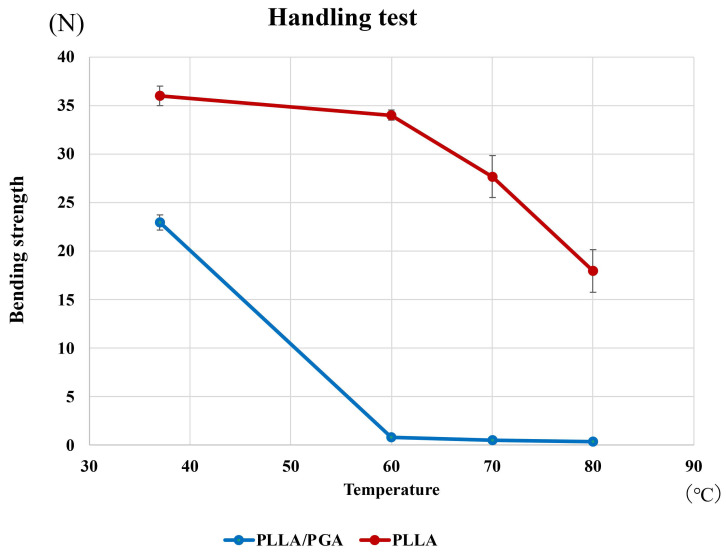
Comparison of PGA-co-PLLA and PLLA in handling test.

In the handling test, PGA-co-PLLA exhibited a noticeable decrease in bending strength when the temperature exceeded 60 degrees, demonstrating high handling performance (Figure 7).

### Biomechanical loading evaluation

In the biomechanical indentation strength tests with anterior loading, there were no significant differences between PGA-PLLA and PLLA at maxillary segment displacements of 0, 3, or 5 mm.

**Figure 8:**
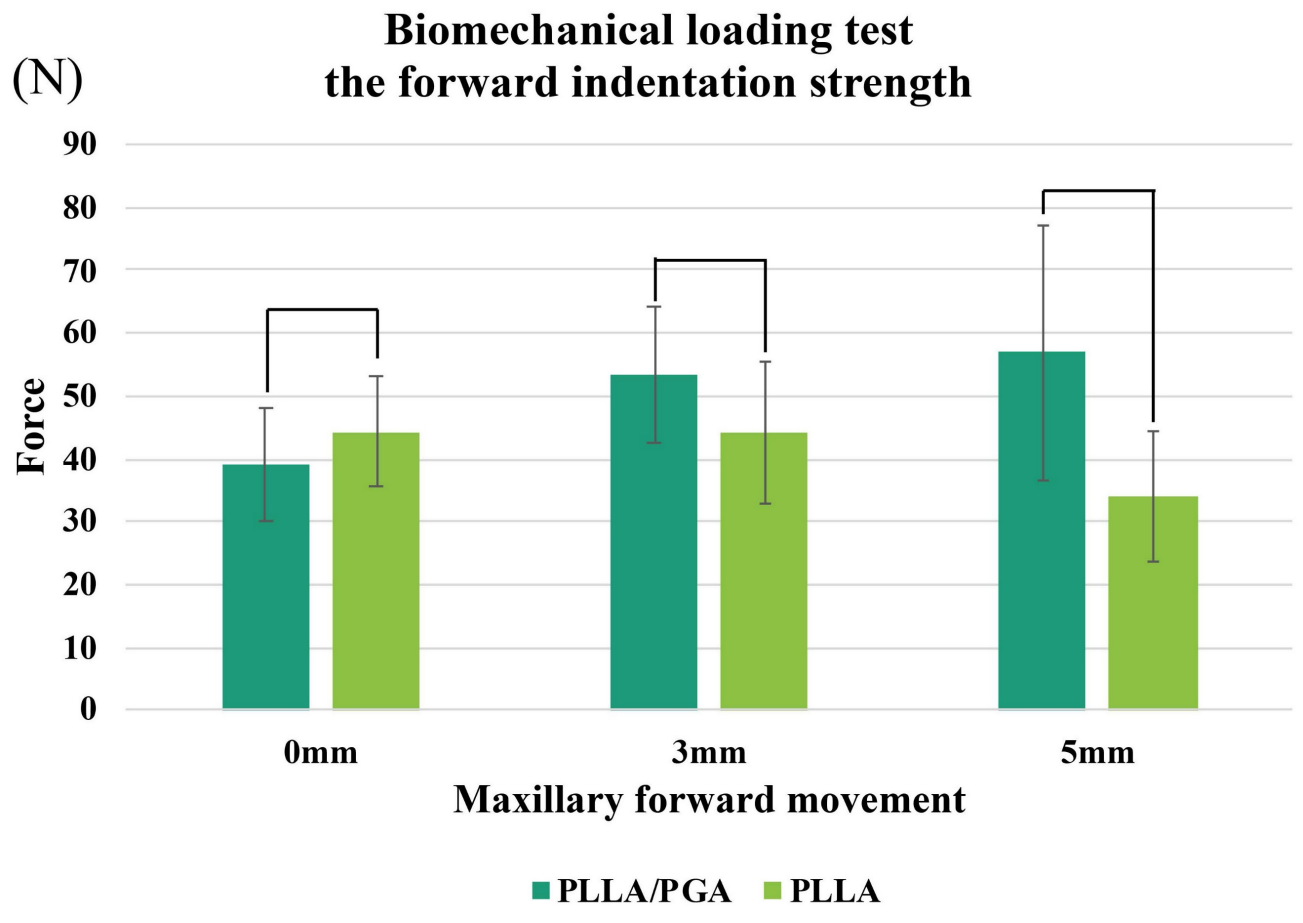
Comparison of PGA-co-PLLA and PLLA in biomechanical tests of forward compression strength at 1mm compression.

At 0 mm displacement, PGA-co-PLLA had 88.2% of the strength of PLLA; however, at 3 and 5 mm, these estimates were 120.7% and 167.3%, respectively (i.e., PGA-co-PLLA had a higher strength) (Figure 8).

**Figure 9:**
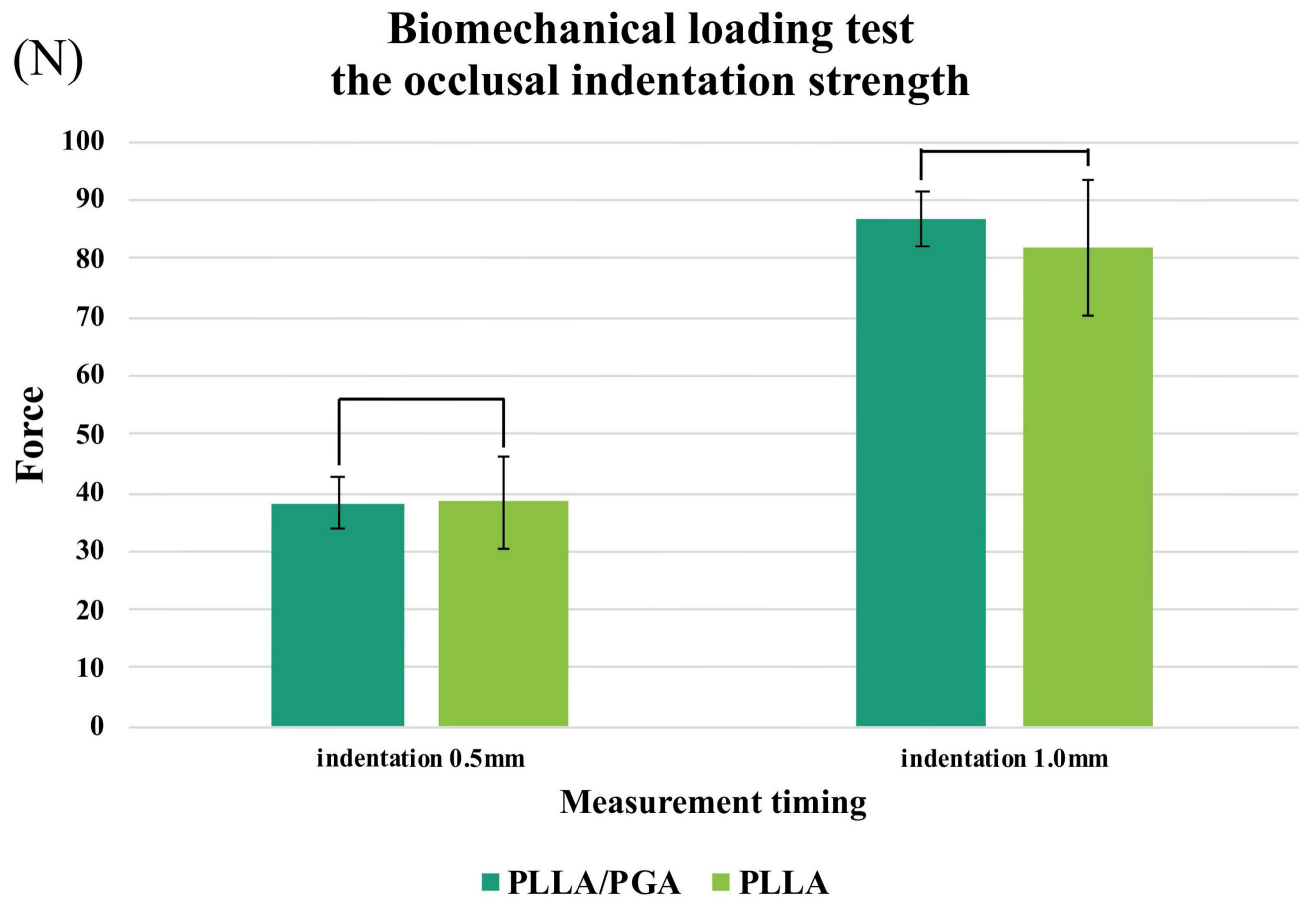
Comparison of PGA-co-PLLA and PLLA in occlusal indentation strength tests.

In a biomechanical indentation strength test using a load from the occlusal surface on a model with 2 mm bone separation and loss of continuity, no significant difference between PGA-co-PLLA and PLLA was observed at either 0.5 or 1.0 mm. Additionally, at 0.5 mm push-in, PGA-co-PLLA exhibited 99.7% of the strength of PLLA, and at 1.0 mm, PGA-co-PLLA exhibited the same strength as PLLA (106.3%) (Figure 9).

## Discussion

This is the first in vitro biomechanical evaluation comparing rapidly resorbable PGA-co-PLLA and conventional resorbable PLLA plate systems for bone integration after LF1 osteotomy. In basic physical strength tests, the rapidly resorbable plate was significantly weaker than the PLLA plate. In contrast, in biomechanical strength tests for LF1, there were no significant differences in strength between anterior and occlusal indentations. These results suggest that the difference in physical strength between PGA-co-PLLA and PLLA is small for applications as a bone integration material after LF1 osteotomy.

The mechanical properties of lactic acid-based polymers can vary widely, from soft and elastic plastics to hard and strong materials 10. Applications of these polymers as an osteosynthetic material in the human body require good mechanical properties. For this reason, semi-crystalline PLLA is traditionally used. PLLA is a biocompatible and bioabsorbable polymer; however, it can be improved by mixing with other materials to promote decomposition and absorption or impart bioactivity^11^. However, the dispersion of other materials reduces the strength of PLLA. In this study, the strength of PGA-co-PLLA was lower than that of PLLA alone based on all basic physical parameters, including tensile, bending, and indentation strengths.

A study of time-dependent alterations in bite force subsequent to LFI orthodontic surgery revealed values of 97.6 N at 1 month, 206.9 N at 3 months, and 257 N at 6 months. Sugiura et al. 12 elucidated that the apex of stress levels on miniplate surfaces materializes within 2-4 weeks after surgical intervention, thus establishing a benchmark of 97.6 N. The occlusal push-in test revealed strengths of 81.7 N for PLLA and 86.9 N for PGA-co-PLLA. This test replicated a clinical scenario lacking bone continuity, and our findings indicated that the plate alone was marginally insufficient with respect to strength. These results have significant implications for clinical applications. In cases where complete bone continuity is absent during maxillary movement, implementation of measures to assist with load bearing may be required. The use of bone grafts or intermaxillary fixation in the affected area is recommended to establish continuity.

Interestingly, despite the lower basic physical strength of the PGA-co-PLLA in this study, the biomechanical strength of the LF1 model did not differ significantly from that of the PLLA model. At a 1 mm indentation, PGA-co-PLLA exhibited higher strength. PLA is an extremely brittle material with an elongation at break of < 10% 13. The toughness of PLLA can be enhanced by copolymerization with other materials 14. The increased strength observed at a 1-mm indentation in the occlusal biomechanical evaluation in this study can be attributed to the physical properties of the plate.

In an anterior weight-bearing analysis followed the methodology outlined in a previous study 9, anterior translations of 0, 3, and 5 mm were used to simulate clinical scenarios. However, no significant differences were observed between the PLLA and PGA-co-PLLA groups. Notably, the strength of PGA-co-PLLA increased with greater translation. This variance in strength reflects the distinct physical properties of the PLLA and PGA-co-PLLA plates. This seemingly counterintuitive result may be attributed to the viscoelastic properties of the PGA-co-PLLA material. Under conditions of larger displacement, the higher deformability of the PGA-co-PLLA plate may allow more uniform stress distribution, thereby preventing localized failure.

This study represents the first investigation of the fundamental physical and biomechanical characteristics of PLLA and PGA-co-PLLA plates in LF1. Furthermore, basic physical properties were assessed to evaluate their ease of handling. PGA-co-PLLA demonstrated superior operability. Although the use of custom-made plates has become increasingly feasible in recent years^15^, this practice remains uncommon for absorbable materials. Intraoperative bending is required; however, the maxilla has a highly intricate morphology. Thus, the ability to easily bend plates along a template is crucial 16. Moreover, biomechanical evaluation revealed no disparity in strength between PLLA and PGA-co-PLLA. We expect these results to guide plate selection in future clinical practice. Although this study focused on the comparison of basic and biomechanical properties at a single time point, it did not include time-dependent degradation testing. The rate at which PGA-co-PLLA loses its mechanical integrity over time compared to PLLA remains an important factor for clinical application, particularly regarding fixation stability during the bone healing period. Future studies should therefore investigate the degradation kinetics and residual strength retention of these materials to provide a more comprehensive understanding of their long-term biomechanical behavior.

This study had some limitations that should be acknowledged. One is the limited variation in the direction and magnitude of the movement considered. The direction of maxillary bone movement can vary, and partial bone continuity may occur in certain cases. Ideally, all of these scenarios should be addressed, which is not feasible in practice. Therefore, we assessed a model that represents typical movement directions without bone continuity, which is the most clinically uncertain scenario.

Another limitation is that the evaluation was based solely on the device used in this study; specifically, we did not compare the PLLA plates with metal plates. Although clear differences between metal and PLLA plates after LF1 have not been reported, further investigation is needed.

Overall, this study provides the first in vitro comparison of the fundamental physical and biomechanical properties of rapidly resorbable PGA-co-PLLA and conventional resorbable PLLA plate systems for osteosynthesis following LF1 osteotomy. The basic physical strength tests revealed that the PGA-co-PLLA plate was significantly weaker than the PLLA plate, although it was remarkably easier to handle. Conversely, the biomechanical strength test of LF1 demonstrated no significant difference in strength between the two plates, either in anterior or occlusal indentations. These findings provide novel evidence for the selection of resorbable plates in clinical practice.
